# MORITS: An improved method to predict peptides from heterologous proteins that are recognized by the same T-cell receptor

**DOI:** 10.1038/s41598-024-58350-x

**Published:** 2024-04-08

**Authors:** Matthias Bruhn, Moritz Spatz, Ulrich Kalinke

**Affiliations:** 1 Institute for Experimental Infection Research, TWINCORE, Centre for Experimental and Clinical Infection Research, a joint venture between the Helmholtz Centre for Infection Research and the Hannover Medical School, Hannover, Germany; 2 Cluster of Excellence RESIST (EXC 2155), Hannover Medical School, Hannover, Germany

**Keywords:** Computational biology and bioinformatics, Immunology

## Abstract

Antigen-specific priming of T cells results in the activation of T cells that exert effector functions by interaction of their T-cell receptor (TCR) with the corresponding self-MHC molecule presenting a peptide on the surface of a target cell. Such antigen-specific T cells potentially can also interact with peptide-MHC complexes that contain peptides from unrelated antigens, a phenomenon that often is referred to as heterologous immunity. For example, some individuals that were pre-immunized against an allergen, could subsequently mount better anti-viral T-cell responses than non-allergic individuals. So far only few peptide pairs that experimentally have been shown to provoke heterologous immunity were  identified, and available prediction tools that can identify potential candidates are imprecise. We developed the MORITS algorithm to rapidly screen large lists of peptides for sequence similarities, while giving enhanced consideration to peptide residues presented by MHC that are particularly relevant for TCR interactions. In combination with established peptide-MHC binding prediction tools, the MORITS algorithm revealed peptide similarities between the SARS-CoV-2 proteome and certain allergens. The method outperformed previously published workflows and may help to identify novel pairs of peptides that mediate heterologous immune responses.

## Introduction

The immune response against a certain antigen can differ strongly between single individuals with regard to the quality and quantity. Many factors can influence human immune responses and another layer of complexity is added by the fact that an adaptive immune response against one pathogen may influence the outcome of a subsequent infection with a second, unrelated pathogen. This concept is also known as heterologous immunity^1^. Antibodies that cross-neutralize closely related viruses are common^2^, but even antibodies that cross-neutralize viruses from different species were described^3^. Also T cells are prone to mediate heterologous immunity. If two peptides are similar enough to be recognized by the same T-cell receptor (TCR), T cells that were induced earlier against one antigen could induce a fast recall immune response against another antigen. Such patterns have indeed been observed for a number of related and unrelated viral pathogens^4^. For example, cross-reactivity between peptides from the hepatitis C virus NS3 protein and influenza A virus neuraminidase was reported^5^. These peptides share a surprisingly high degree of sequence similarity and are recognized by the same TCR^5^. Such cross-reactive T-cell responses are not always protective, but can also cause enhanced pathology as observed for a cross-reactive, highly similar epitope pair that is found in lymphocytic choriomeningitis virus and pichinde virus^6^. Another possible consequence of TCR cross-reactivity may be the infection-provoked onset of autoimmunity through a mechanism termed molecular mimicry^7^.

In addition, TCR cross-reactivity theoretically can occur between pathogens and environmental antigens such as allergens. Evidence for protection from asthma through a previous influenza infection and/or immunization was found in a mouse model and it was speculated that sequence similarities in certain epitopes of influenza A virus and house dust mite allergens account for the effect^8^. More recently, the hypothesis was raised that such sequence similarities can affect the susceptibility for SARS-CoV-2 infection. In an association study that was carried out during the early COVID-19 pandemic, it was found that allergic asthma patients have a lower risk to develop severe COVID-19 infection than individuals with non-allergic asthma^9^. Based on this observation, another study proposed an in silico screening method for identification of potentially cross-reactive T-cell epitopes that are detected in SARS-CoV-2 and environmental allergens^10^. The authors presented a list of short peptide sequences with a certain degree of similarity, which they predicted to be presented on the same major histocompatibility complex (MHC) allele and therefore potentially could be recognized by the same TCR. The proposed bioinformatics workflow is based on BLAST^11^ alignments. Another study also used BLAST in order to identify sequence similarities between SARS-CoV-2 and pathogenic bacteria^12^. This alignment algorithm tolerates amino acid deletions and insertions. For this purpose, this is not a desired feature, because it is very unlikely that a TCR still cross-recognizes peptides that align only by creation of gaps, considering that the spatial position of the respective amino acids within the peptide will be very different in such cases. Furthermore, BLAST does not allow prioritizing certain positions within an amino acid sequence and therefore cannot take into account that the orientation of the amino acids within the peptide may be crucial to mediate cross-reactivity.

Structural data as well as MHC binding studies revealed that some peptide positions within the binding core are deeply buried in the binding groove of the MHC. In contrast, amino acids at the remaining positions are not strongly involved in MHC binding, but instead are directed towards the outside. As a consequence, such residues are more accessible for the TCR and are referred to as T-cell exposed motifs (TCEMs). While for MHC I the TCEM 1 is located at positions 4–8 of a 9-mer, there are two possible TCEM patterns for MHC II when considering the central 9-mer, TCEM 2a at positions 2, 3, 5, 7 and 8 and TCEM 2b at positions -1, 3, 5, 7 and 8^13^.

Here, we developed a user-friendly tool that allows screening of large lists of proteins for short peptide sequences that show identical amino acids in the TCEMs, while allowing mismatches in the non-TCEM positions. We combined this approach with MHC I and MHC II binding predictions in order to identify peptide candidates that, in addition to sharing the TCEM, are predicted to bind strongly to the same MHC alleles. When applied to the same dataset as used by Balz et al.^10^, we found a different set of allergen-derived peptides that might mediate heterologous immunity towards SARS-CoV-2. The peptides we identified showed high similarity and therefore more likely cause heterologous immunity than other ones found in the existing literature. Our refined method can form the basis to develop new hypotheses in the research field of heterologous T-cell immunology, which currently transitions into a new era of computational approaches and high-throughput validation studies^14^.

## Results

### Development of the MORITS tool

**Figure 1:**
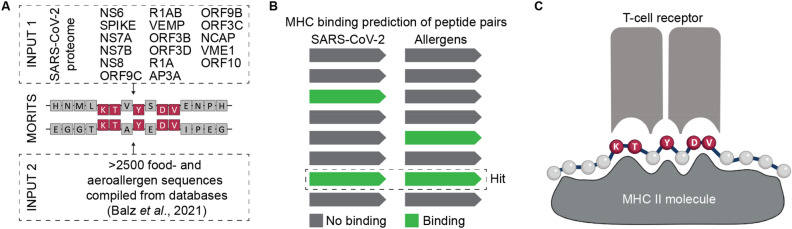
Prediction of peptide sequences that are presented by the same MHC allele and that potentially interact with the same TCR. (**A**) Using the MORITS algorithm, the SARS-CoV-2 proteome was compared to a published set of known allergen proteins in order to identify sequences with shared T-cell exposed motif (TCEM, highlighted in red). (**B**) MHC binding prediction reveals those sequences, for which both the viral and allergen peptide strongly bind the same MHC allele. (**C**) Schematic depiction of a peptide-MHC II complex, in which only the amino acids in the TCEM (red) interact with the T cell receptor.

To allow fast identification of peptide pairs that are identical in their TCEM, we developed the MHC outward-facing residue identifying tool for sequence alignment (MORITS). It contains options to choose the desired TCEM template (TCEM 1 for MHC I, TCEM 2a and 2b for MHC II) and the extent of deviation from the sequence of the five TCEM residues, ranging from identical (100%) to one mismatch (80%). The latter option is less stringent, because only 4 out of 5 TCEM residues coincide. After the alignment of the two input files (for details see Methods section), the result file can be saved as a text file for further processing (Fig. 1A). The paired and numbered result files were subsequently used to predict the binding to reference MHC allele sets using external, already available MHC binding prediction tools (MORITS does not include this function). Peptide pairs in which both the viral and the allergen peptide are predicted to bind to the same MHC allele were filtered (Fig. 1B). Such peptide pairs were handled as potential candidates to mediate heterologous T-cell responses (Fig. 1C).

### Predicted MHC I binding peptides share similarities with animal, plant and fungal allergen sequences

The MORITS tool identified a total of 1584 9-mers, which show 100% similarity along the 5 amino acid TCEM 1 between one of the SARS-CoV-2 proteins and one of the allergens investigated previously^10^. Those hits were used to predict the MHC I binding. To this end, they were filtered for peptide pairs, in which both the peptide from SARS-CoV-2 as well as the allergen peptide were predicted to bind strongly to the same MHC I allele. Nine peptides passed the filtering (Table 1). Five different SARS-CoV-2 proteins were identified to show some similarity with one of the allergens, and the identified allergens are from various different animal, plant and fungal sources.

**Table 1:**
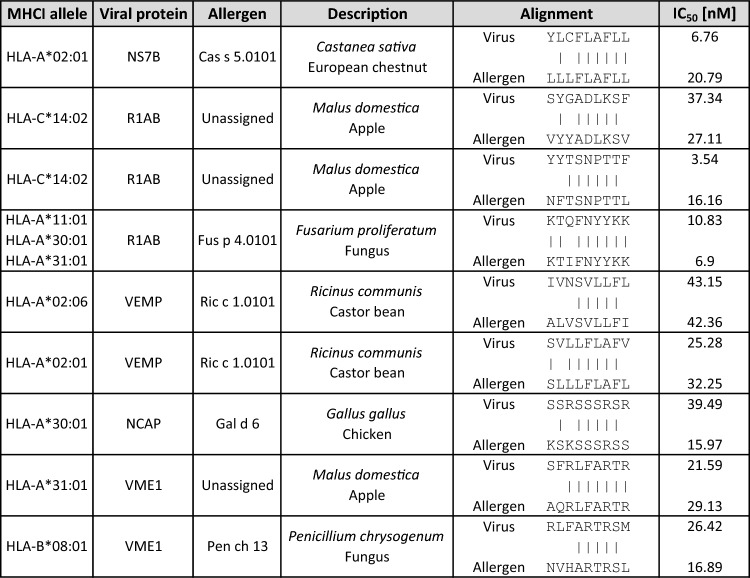
MHC I binding prediction of SARS-CoV-2 and allergen derived peptides.

The chosen 9-mers possess a similarity of at least 5 amino acids in the TCEM (position 4–8). The results were filtered for an IC_50_ of less than 50 nM.

### Similarities in predicted MHC II binding between SARS-CoV-2 antigens and allergens are enriched for the replicase polyprotein 1ab

For the two possible TCEM 2, a total number of 3240 similar regions were identified by the MORITS tool. After binding prediction to MHC II, twelve hits passed the filtering (Table 2). Eleven of those concerned the viral protein R1AB and one the spike protein. The counterpart allergens originated from different plant, animal and fungus sources. High similarity was detected between R1AB and Sor h 1 and Zea m 1, indicating that the corresponding sequences are conserved in *Sorghum halepense* and *Zea mays*.

**Table 2:**
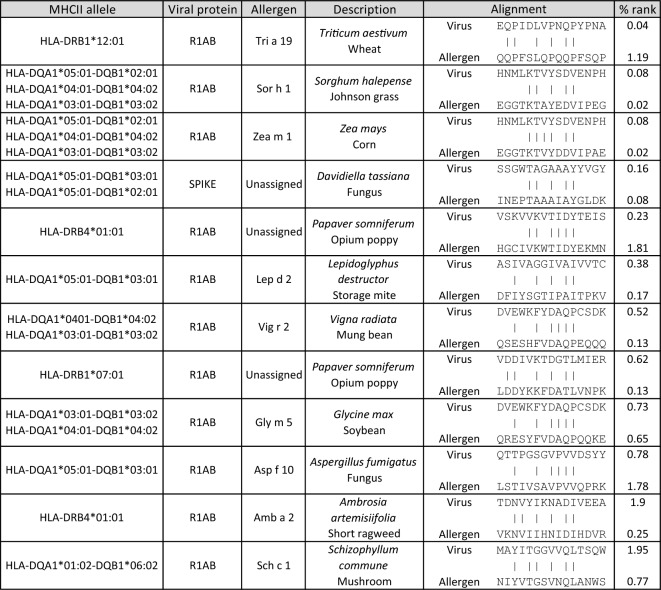
MHC II binding prediction of SARS-CoV-2 and allergen derived peptides.

The chosen 15-mers possess a similarity of at least 5 amino acids in the TCEM (either positions 5, 6, 8, 10 & 11 or 3, 6, 8, 10 & 11). The results were filtered for a percentile rank of less than 2.0.

### MORITS outperforms existing alignment strategies

**Figure 2:**
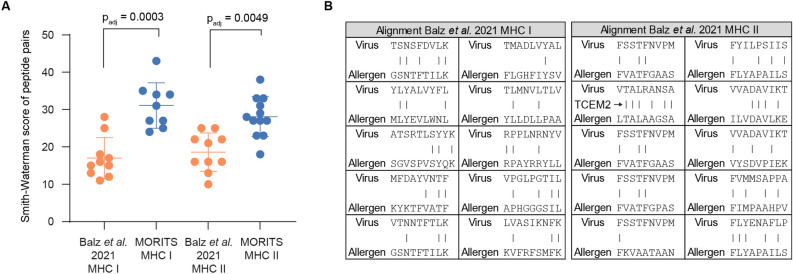
The MORITS algorithm outperforms previously published strategies for identification of candidates for heterologous T-cell immunity. (**A**) Smith–Waterman alignment scores were calculated for peptide pairs identified in this study and in Balz et al.^10^. The whiskers indicate the mean and SD for each group. Statistical (nonparametric) tests were performed and the adjusted *p* value is indicated. (**B**) The top candidate peptide pairs from Balz et al.^10^ were aligned and investigated for their similarity between the peptides derived from virus and allergen. Only one pair fulfills the TCEM criteria and is highlighted with an arrow.

In order to assess how the MORITS algorithm performs in comparison with earlier published strategies that were based on BLAST alignments, we used the results presented by Balz et al.^10^ as a benchmark. Overall, not a single putative allergen peptide was shared between the study by Balz et al*.* and our study. The most promising candidate pairs identified by the MORITS algorithm had higher similarity between virus and allergen peptides than the previously published candidates, as indicated by the significantly higher alignment score (Fig. 2A). Investigating the distribution of amino acid similarities over the length of the identified peptides, not a single MHC I peptide pair and only one out of ten MHC II peptide pairs from Balz et al. fulfilled the TCEM criteria (Fig. 2B), while all peptide pairs identified in this study showed identical TCEM residues (Tables 1, 2). In conclusion, the MORITS algorithm reveals potential candidates for heterologous immunity that share all amino acid similarities at the relevant positions while at the same time significantly outperforming existing pipelines in terms of revealing overall sequence similarity.

### Similarities between SARS-CoV-2 and common cold human coronaviruses

Our predictions of potentially cross-reactive T-cell epitopes have not been validated experimentally, yet. In order to address whether the results generated by MORITS can help to formulate biologically relevant hypotheses, we tested the method on an experimentally validated set of peptide sequences (HCoV-R129) described by Mateus et al.^15^. In this study, the authors tested a set of 129 peptides derived from the common cold human coronavirus strains 229E, HKU1, NL63 and OC43 for cross-reactivity to SARS-CoV-2 in pre-pandemic samples. A subset of the SARS-CoV-2 peptides that was specifically recognized by CD4^+^ T cells, cross-reacted with at least one strain of the common cold human coronavirus. We used the MORITS pipeline on this dataset and predicted 10 hits, out of which 7 ones are among the experimentally validated cross-reacting peptides (Table 3). These data suggests a high resolution of the MORITS algorithm in predicting cross-reactive T-cell epitopes with a relatively low rate of false positive results.

**Table 3:**
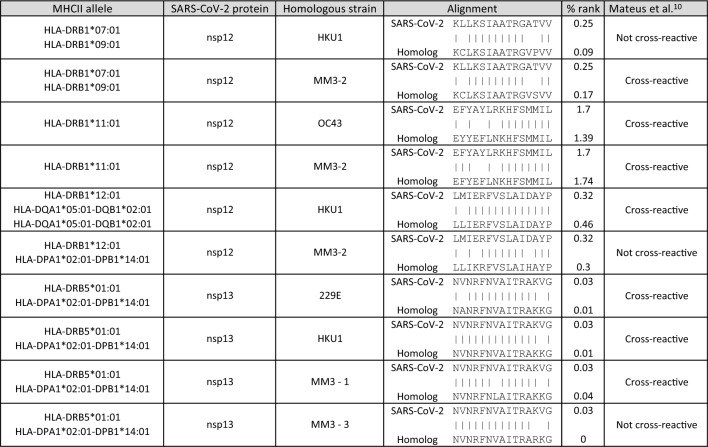
MHC II binding prediction of SARS-CoV-2 and human coronavirus-derived peptides.

The chosen 15-mers possess a similarity of at least 5 amino acids in the TCEM (either positions 5, 6, 8, 10 & 11 or 3, 6, 8, 10 & 11). The results were filtered for a percentile rank of less than 2.0.

## Methods

### Retrieval of protein sequences

For the SARS-CoV-2 protein sequences, the reference proteome UP000464024 was retrieved from the Uniprot database in fasta format. A recently published listing of different food and air allergens^10^ was downloaded and a single fasta file was created, which contained 2597 entries.

### High throughput protein sequence alignment in TCEM 1 and TCEM 2 mode

The input fasta files were converted into csv files and compared to each other with the in house developed MHC outward-facing residue identifying tool for sequence alignment (MORITS). The MORITS tool was developed for the purpose of sequence alignments taking the TCEM templates into account. The C # programming language was used to develop the tool and a Windows GUI was created for user friendliness. Visual Study Community 2019 served as the programming environment.

The corresponding csv files were selected via a file dialog in the Windows GUI. The files are read into the program and converted to string arrays. Then the desired TCEM templates and the matching precision was selected. During this matching process, the algorithm starts by reading the first 15 characters of sequence 1. Afterwards the first 15 characters of sequence 2 are aligned and it is tested if the characters are equal over the important positions of the TCEM template. In the next step, the reading frame of sequence 2 moves one position forward and the characters are tested again. After all possible positions of sequence 2 were tested for the relevant positions of the first 15 characters of sequence 1, the reading frame of sequence 1 shifts one position forward and the process starts again. If there is a match in the selected template with 80% or 100% (depending on the user’s choice) the match is going to be displayed in the bottom text frame and in the .txt output file. With this method all possible alignments between the two selected sequences will be tested on all positions for a match on the positions relevant for the selected TCEM. TCEM 1 and TCEM 2 results were saved separately and the tool was set to 100% sequence similarity. The results of each alignment were imported into Microsoft Excel. For each TCEM 1 and TCEM 2 results, two fasta files were created by saving tab-separated tables using Microsoft Excel and converting them to fasta format using the tab to fasta converter on https://sequenceconversion.bugaco.com. One fasta file contained the identified hits for the viral peptides, and the other one contained the corresponding hits for the allergen peptides.

### MHC binding prediction

The MHC binding prediction was performed for 27 MHC I molecules and 27 MHC II heterodimers according to the reference panels offered by IEDB in order to cover a high percentage of the human population^16,17^. The alleles are listed in Table S1.

For MHC I binding prediction, a standalone version of the IEDB binding prediction tool was installed locally on a linux server (http://tools.iedb.org/mhci/download/). The TCEM 1 fasta files containing the 15-mers for SARS-CoV-2 and allergen hits were shortened down the middle 9-mer core^13^ using the Excel MID function. Virus and allergen sequences were processed each separately by the tool using the IEDB recommended 2020.09 (NetMHCpan EL 4.1) settings. The results were imported and filtered in Microsoft Excel.

For MHC II binding prediction, the tool netMHCIIpan 4.0^18^ was used as standalone linux installation. The 15-mer TCEM 2 result files for SARS-CoV-2 and allergens were processed individually and the binding prediction results were imported and filtered in Microsoft Excel.

Filtering of the results was performed using Microsoft Excel in order to derive peptide pairs which are (1) similar in their TCEM and (2) predicted to bind on the same HLA allele. Duplicates that had the same SARS-CoV-2 peptide and the same or a highly similar allergen counterpart (caused by multiple submissions of the same protein in the database with minor sequence differences) were removed. If the same pair was identified for multiple alleles, those are reported together, indicating only the stronger binding prediction as IC_50_ or percentile rank, respectively. The allergen IUIS codes were retrieved from AllergenOnline^19^ or, when no entry was present there, from Uniprot.org.

A cutoff of IC_50_ < 50 nM for MHC I and of percentile rank < 2.0 for MHC II was used to select for the strongest binding predictions. This threshold can be modified to lead to a more or less stringent filtering.

### Calculation of alignment scores

To assess the similarity between peptide pairs identified in this study and in previous publications, the Smith–Waterman algorithm^20^ was used via the EMBOSS^21^ Water web server on default settings. The results were plotted using GraphPad Prism 8.4.2 and statistical analysis was performed using the nonparametric test with correction for multiple hypothesis testing. Additionally, the alignments were visualized by using Microsoft Excel with lines indicating an identical amino acid.

### Performance

The alignment speed of MORITS is proportional to the size of the protein sequences used as input files. We tested different input file sizes on a standard office laptop (Windows 10, Intel Core i7 2.8 GHz, 16 GB RAM) and the program processed comparisons between the SARS-CoV-2 proteome and the human cytomegalovirus (HCMV) proteome in less than 2 min, compared SARS-CoV-2 versus the allergen database used in this study in 13 min and SARS-CoV-2 versus the entire human proteome completed in around 2.5 h.

## Discussion

In this study, we developed a tool that identifies similar peptides derived from different proteins that are presented by identical MHC molecules. Importantly, this tool takes into account T cell exposed motifs (TCEM). Combined into a pipeline with established MHC binding prediction tools such as netMHCIIpan^18^, our tool can be used to rapidly identify candidate peptides that potentially are involved in heterologous T-cell responses. We investigated whether our tool would reveal the same collection of similar peptides derived from the SARS-CoV-2 proteome and common environmental allergens, as was proposed in a recent publication^10^. To our surprise, an entirely different set of peptide pairs was identified by our tool, and our peptide candidates were not reported in prior literature. The peptide pairs identified in our study show higher similarities between virus and allergen-derived peptides, while still being predicted to be presented on the same MHC molecules. Furthermore, the peptide pairs presented by Balz et al.^10^ often have identical amino acid sequences at positions, which are unlikely to be exposed towards the TCR. Therefore, candidate pairs identified with the help of the MORITS algorithm have a high likelihood to be candidate peptides that account for heterologous T-cell responses.

In contrast to B-cell epitopes, which mostly consist of three-dimensional surface structures, T-cell epitopes consist of a linear peptide sequence, which is bound by an MHC molecule. It has long been discussed whether a given TCR is specific only for one single peptide that is presented by the appropriate self-MHC, or whether the TCR:peptide-MHC interaction is more promiscuous in the sense that one TCR can interact with several similar peptides presented by the same MHC molecule. The latter is more likely to be the case because the human body only possesses a limited number of T cells and the sheer number of existing foreign peptides presumably exceeds the number of available TCR clones^22,23^. Furthermore, a principle that was proposed is that not all nine amino acids are similarly important for the interaction with the TCR. While some peptide residues within the peptide-MHC complex are rather hidden in the binding groove, others are more accessible to the TCR^13^. The five residues presumably most exposed to the outside form the TCEM^13^. Of note, the concept of the TCEM may be an oversimplification and does not capture the whole complexity of peptide binding to MHC, considering that different allotypes may differ in their anchor positions and special cases like reverse binding of peptides on HLA-DP were reported^24^.

The likelihood that two 9-mer peptides derived from two unrelated proteins are identical is 1:512 billion (1:20^9^), making the primary sequence of any T-cell epitope exceptionally specific. Following the concept that not all residues are equally relevant for the interaction with the TCR, but the five most exposed amino acids form the TCEM, the specificity of a T-cell epitope is reduced^13^. The likelihood that two unrelated 9-mers are identical in their TCEM is 1:3.2 million (1:20^5^), providing that they are both presented by the same MHC allele. The numbers indicate that the occurrence of promiscuous TCRs that interact with a combination of one MHC with different peptides is rather realistic, even if entirely unrelated proteins such as virus antigens and environmental allergens from plants, animals and fungi are considered. A systematic assessment of which TCRs in the human repertoire react with which related and unrelated antigens currently cannot be carried out due to the immense number of peptides to be tested for every TCR. Due to the expected low frequency of such events^25^, an in silico pre-screen to identify peptide similarities that could result in TCR cross-reactivity is a feasible option. Tools such as MHC binding prediction and the MORITS algorithm may support future research for the discovery of heterologous T-cell responses in various settings. Another possible aspect for the clinical relevance of heterologous T-cell responses may be the specificity of T-cell-based therapies. CrossDome, a tool which predicts cross-reactivity in this context was recently described^26^.

Currently, the alignment in MORITS is limited to 15-mers for MHC II and 9-mers for MHC I, which were selected on the basis of the TCEM definitions described previously^13^. In reality, different lengths of peptides may be presented on the MHC molecules and future studies need to address the influence of the peptide length on TCR binding. Considering that the vast majority of cross-reactivity may be attributable to the central portion of the peptide (Fig. 1C), we estimate this limitation to be of low influence on the overall outcome of the prediction.

Another limitation of our study is the lack of experimental validation. Rather than similarities of primary peptide sequences (which can easily be assessed by computational methods), structural traits of peptide-MHC interactions might be relevant to confer heterologous T-cell responses (which are not considered by the MORITS algorithm). At this moment, this kind of interaction is very difficult to predict and even disparate peptides with little sequence similarity potentially can cause heterologous immunity^27,28^. A tool aiming to take this structural aspect into account is MatchTope^29^.

Furthermore, it remains unknown whether TCR cross-reactivity is a directional phenomenon, i.e., whether T cells primed against one antigen also recognize heterologous antigens, but not the other way around. Therefore, we estimate that in silico prediction methods are intrinsically error-prone and only a fraction of the peptide pairs identified by MORITS would be recognized by the same T cells. Correspondingly, our pipeline is suited to reduce the number of potentially heterologous peptides that in the next step can be further analyzed experimentally.

We attempted to estimate the sensitivity and specificity of MORITS on an experimentally validated set of peptides derived from SARS-CoV-2 and human common cold coronaviruses published previously^15^. 70% of the hits revealed by MORITS were experimentally proven to cross-react on CD4 T cells (see Table 3). On the other hand, several truly cross-reactive peptide pairs were missed by MORITS, indicating an imperfect sensitivity of the method. However, these results should be interpreted with caution, since the peptide sequences used for this comparison were homologs between SARS-CoV-2 and other strains of coronaviruses, which share a high degree of sequence similarity. In contrast, truly heterologous T-cell responses occur due to coincidental sequence similarities such as observed here between SARS-CoV-2 and environmental allergens. In these instances, we estimate the specificity and sensitivity of MORITS to be lower.

## Data availability

The SARS-CoV-2 reference proteome was retrieved from Uniprot under accession number UP000464024. The list of different food and air allergens was retrieved from Balz et al*.*^10^. An executable version of the MORITS algorithm, the underlying raw code in C# format as well as the exemplary input data used in this study are available under https://github.com/memumab/MORITS.

### Supplementary Information

Supplementary Table S1.
